# Willingness and Perceptions Regarding COVID-19 Vaccine Booster Dose in Pakistani Vaccinated Population: A Cross-Sectional Survey

**DOI:** 10.3389/fpubh.2022.911518

**Published:** 2022-06-30

**Authors:** Abdul Moeed, Hala Najeeb, Arisha Saleem, Muhammad Sohaib Asghar, Hania Mansoor Rafi, Abdullah Khan Khattak, Zoha Bilal, Binyam Tariku Seboka

**Affiliations:** ^1^Department of Internal Medicine, Dow University of Health Sciences, Karachi, Pakistan; ^2^Department of Internal Medicine, Dow University Ojha Hospital, Karachi, Pakistan; ^3^Department of Internal Medicine, Aga Khan University, Karachi, Pakistan; ^4^School of Public Health, Dilla University, Dilla, Ethiopia

**Keywords:** willingness, perceptions, COVID-19, COVID-19 vaccine booster, vaccinated population, vaccine booster dose

## Abstract

**Objectives:**

This study was conducted to evaluate COVID-19 vaccine booster dose willingness and identify predictors and factors of willingness and hesitance in the vaccinated population of Pakistan.

**Methods:**

A cross-sectional web-based survey was undertaken between January and February 2022 to highlight the public perceptions regarding the COVID-19 booster dose and evaluate the willingness to get the additional dose. Demographic information and booster dose willingness were recorded through the questionnaire. Additionally, a 5-point Likert scale was employed to explore fears and beliefs regarding COVID-19 vaccinations. Univariate and multivariate regression was performed to identify booster dose willingness and hesitance factors.

**Results:**

Of the 787 respondents, 69.6% were females, 75.3% fell in the 18–30 years age group, 53.5% were university students or had a Bachelor's degree. Overall, a 77.8% booster dose willingness was reported. Participants showed absence or low fear levels associated with a booster dose (47.3%). 60.1% agreed it was safe to receive an additional vaccine dose, with 44.1% agreeing that boosters are effective against coronavirus variants. Independent predictors of willingness included the absence of comorbidities, whereas not being willing to pay for the booster dose was a predictor of hesitance.

**Conclusion:**

This study showed a suboptimal willingness level of booster dose uptake among the vaccinated Pakistani population. Public health policymakers must undertake necessary awareness campaigns to strategize vaccination drives and dispel myths.

## Introduction

Since its emergence in December 2019 in Wuhan, China, the coronavirus disease (COVID-19) has plagued the entire world, wreaking havoc on the public healthcare system (1). With approximately 433 million active infections and 5.94 million deaths (2), the severe acute respiratory syndrome coronavirus-2 (SARS- CoV-2) outbreak has been the greatest public health hazard posed to humanity since the discovery of human immunodeficiency virus (HIV) 40 years ago (3). This necessitated the development of a COVID-19 vaccine. With the administration of the ChAdOx1-S/nCoV-19 (AstraZeneca) vaccine, on January 4, 2021, the United Kingdom became the first country to commence the COVID-19 vaccination campaigns, with other countries soon following suit (4). To combat the virus's spread, the government of Pakistan, along with having published many public health guidelines and preventative measures, also made vaccination available for the public (5). The vaccination was subsequently rolled out in a diminishing age sequence or decreasing risk of acquiring infection and by the 25th of August 2021, 6.3% of Pakistan's population had been fully vaccinated, with 10.8% having been partially vaccinated (5). The percentage of fully vaccinated population later increased to 36% by 25 January 2022 with provision of over 1.7 billion doses (6), which included COVID-19 vaccines from Chinese Sinopharm, Sinovac, CanSinoBio, Sputnik V (Gam-COVID-Vac), mRNA-1273 (Moderna), and ChAdOx1-S/nCoV-19 (Astra Zeneca) have been administered (7).

Short-term clinical trial results of various COVID-19 vaccines indicated their potency against symptomatic SARS-CoV-2 infection, later supported by the first post-authorization, real-world observations (8–10). However, substantial evidence confirms that their efficacy declines over time, and the relative risk decrease was determined to be 88% at ≤1 month after the second dose, reduced to 47% at ≥5 months (11). Decreasing levels of serum anti-spike IgG in the months following the second dose and the prevalence of highly infectious novel SARS-CoV-2 variants such as Omicron may be responsible for the declining effectiveness (12, 13). Since the effectiveness of some vaccines diminishes with time, vaccine boosters are additional vaccine doses that must be administered to further protect against infection (14). Because the durability of immunity against the newer and more infectious variants is unclear, infectious disease experts have thoroughly analyzed the necessity for booster shots for specific vulnerable groups and also the general population to enhance immunity (15). In Pakistan, Sinopharm, Sinovac, Pfizer, Moderna and AstraZeneca booster shots have recently been administered (16).

Vaccine hesitancy, defined as a delay in accepting or refusing to get vaccinated, has been designated as one of the top 10 global health issues (17). During the initial vaccination campaigns, varying degrees of vaccine hesitancy were reported across the globe given the diverse social and behavioral influences (18). Fear of the side effects, preconceived notions about the vaccine's ineffectiveness and belief in natural immunity were some of the leading causes of unwillingness to receive initial doses of the COVID- 19 vaccine (19, 20). According to a cross-sectional study conducted in China (15), concerns regarding vaccine safety appeared to be a significant barrier to vaccination uptake, whereas, in Poland, side effects due to previous doses were a leading cause of hesitancy for boosters (21). South Asian countries such as Pakistan, India, Bangladesh, Nepal, Bhutan, Afghanistan, and the Maldives, in particular, are diverse in terms of demographics, religion, and culture and are deeply afflicted by income disparities, poor literacy rates, and health-related issues, which are contributing factors of vaccine hesitancy (17). In addition, false religious beliefs and the dissemination of inaccurate information by influential figures, social media, friends, and family, instill confusion and fear regarding vaccines (6). As a result, vaccine acceptance is substantially lower in Muslim-majority nations, such as Kuwait and Jordan where the vaccine acceptance rates were as low as 23 and 28%, respectively, as reported by Sallam (22). In order to forge herd immunity and reduce COVID-19 morbidity and mortality rate, it is critical to achieve vaccination acceptance. High acceptance rates are required for any vaccination program' to be successful (23), however, little is known regarding booster acceptance in Pakistan or the reasons contributing to reluctance to COVID-19 boosters at present. Therefore, evaluating public acceptance is imperative to devise efficient campaigns to promote COVID-19 booster vaccines among the general public to prevent past catastrophic events, like polio outbreaks, from repeating themselves.

To the best of our knowledge, this is the first study undertaken to quantify booster vaccination acceptance in Pakistan. Through this study, we assess the public's readiness and hesitancy to receive booster shots while bringing to light the wide variety of presumptions surrounding COVID-19 vaccinations. Furthermore, this study aims to reveal the preferred vaccines and the variables that influence people's preferences for instance concerns regarding vaccine safety and their side effects. Data from a self-administered online questionnaire was used to assess people's experience with and their comprehension of COVID-19 vaccines, as well as their willingness to take booster doses. The research of reasons for booster apprehension will aid in the formulation of statistical results to improve booster delivery through focused strategies and enhance confidence in COVID-19 vaccinations.

## Materials and Methods

### Study Design and Study Sample

While designing the study, Strengthening the Reporting of Observational Studies in Epidemiology (STROBE) guidelines (24) for cross-sectional studies were followed. This cross-sectional study was conducted from the 19th of January to the 15th of February 2022, involving the COVID-19 vaccinated people of Pakistan. Vaccinated individuals of all genders aged above 12 years were asked to fill out a Google form that required them to provide consent, so those who did not wish to respond were excluded. The minimum sample size was 384, calculated using Open-EPI (25), considering an anticipated frequency of 50%, with a 5% margin of error and a 95% CI.

### Survey

The primary means of circulation of the form were social media, including WhatsApp, Facebook and Instagram and email, where a link to the form was shared. The questions were written in the English language. The questionnaire consisted of an introductory paragraph explaining that the study aimed to gauge the willingness of the COVID-19 booster dose and the factors associated with its hesitancy. It mentioned those who were involved in the research. Participants were reassured that their anonymity would be maintained and that the results would only be used for research purposes. The outline and layout were adapted from Lai et al. (15) and Rzymski et al. (21). Two senior professors were requested to review the questionnaire to ensure its validity and reliability.

Furthermore, pilot surveys were also run, involving 10 participants to ensure easy readability and concision of the questionnaire. Questions, in the beginning, focused mainly on the sociodemographic of the respondents, their gender, age and level of education, employment status, monthly income, and any disease they were suffering from. The following section was centered around previous vaccinations against COVID-19 and their experience regarding any subsequent symptoms. Participants were then inquired regarding their willingness to get the COVID-19 booster dose and other beliefs and fears related to them using a 5-point Likert scale.

### Statistical Analysis

The Statistical Package for Social Sciences (SPSS) software package (version 25.0) (IBM Corporation, Armonk, NY) was used to analyze the data. Responses were exported from Google Forms to Microsoft Excel 2016, where all the data was assembled. All categorical variables were included in the analysis. For categorical variables, frequencies and percentages were used. Since the data was not normally distributed, we used non parametric tests. Shapiro-Wilk test was carried out to check the normality of the data. Univariate logistic regression was performed to assess the association of sociodemographic variables, fears, previous experiences, already acquired diseases and factors that may improve COVID-19 vaccination booster acceptance among the vaccinated population. Additionally, multivariate regression was performed to calculate the adjusted odds ratio to determine factors associated with booster willingness. A *P*-value obtained through Wald's method of <0.05 was considered statistically significant.

## Results

### Characteristics of the Study Sample

As shown in Table 1, 787 responses were recorded. Nearly three-fourths (75.3%) were 18–30 years old, 69.6% were female, and 60.7% had an undergraduate/bachelor's degree or above education level. The majority of the respondents were unemployed (81.8%) and had monthly household incomes ranging between 50,000 and 100,000 PKR (285- 571 USD) (22.4%). Furthermore, 100% of the respondents had received at least one dose of the COVID-19 vaccine by February 2022. Most of the respondents had received the BioNTech-Pfizer vaccine (32.2%), with Sinovac and Sinopharm closely following suit at 27.7 and 24.6%, respectively. Regarding the health status, only 11.9% reported the presence of any comorbidity, with asthma being the most common (8.3%). 71.2% of the respondents divulged that they were never infected by COVID-19.

**Table 1:** General and demographic characteristics of the study population (*n* = 787).

**Variables**	**Characteristics**	**Frequency**	**Percentage**
Age	<18 years	142	18.0
	18-20 years	259	32.9
	21-30 years	334	42.4
	>30 years	52	6.6
Gender	Female	548	69.6
	Male	239	30.4
Education	Primary/Secondary	39	5.0
	Higher secondary/College	270	34.3
	Undergraduate/Bachelors/University	421	53.5
	Postgraduate/Masters/Ph.D.	57	7.2
Employment	Employed (Government of private)	86	10.9
	Self-employed (Business)	57	7.2
	Unemployed	644	81.8
Household income (per month)	<50,000 PKR	129	16.4
	50,000 to 100,000 PKR	176	22.4
	100,000 to 200,000 PKR	165	21.0
	200,000 to 300,000 PKR	91	11.6
	300,000 to 400,000 PKR	70	8.9
	400,000 to 500,000 PKR	33	4.2
	>500,000 PKR	123	15.6
Status of vaccination	Fully vaccinated	760	96.6
	Partially vaccinated	27	3.4
Vaccine	BioNTech-Pfizer	253	32.1
	Moderna	55	7.0
	AstraZeneca	14	1.8
	Sinopharm	194	24.6
	Sinovac	218	27.7
	CanSino/PakVac	41	5.2
	Sputnik V	7	0.9
	Johnson and Johnson/Janssen	5	0.6
Willingness to receive booster dose	Yes	612	77.8
	No	73	9.3
	Unsure	102	13.0
Previously infected with SARS-CoV-2	Yes	227	28.8
	No	560	71.2
Willingness to pay for booster dose	Yes	434	55.1
	No	353	44.9
Presence of comorbidities	Yes	96	12.2
	*No*	*691*	*87.8*

*PKR, Pakistani Rupee; SARS-CoV-2, severe respiratory syndrome coronavirus 2; Ph.D., Doctor of Philosophy*.

### Self-Perceived Side Effects and Fear Regarding Initial Doses of Vaccine

**Figure 1:**
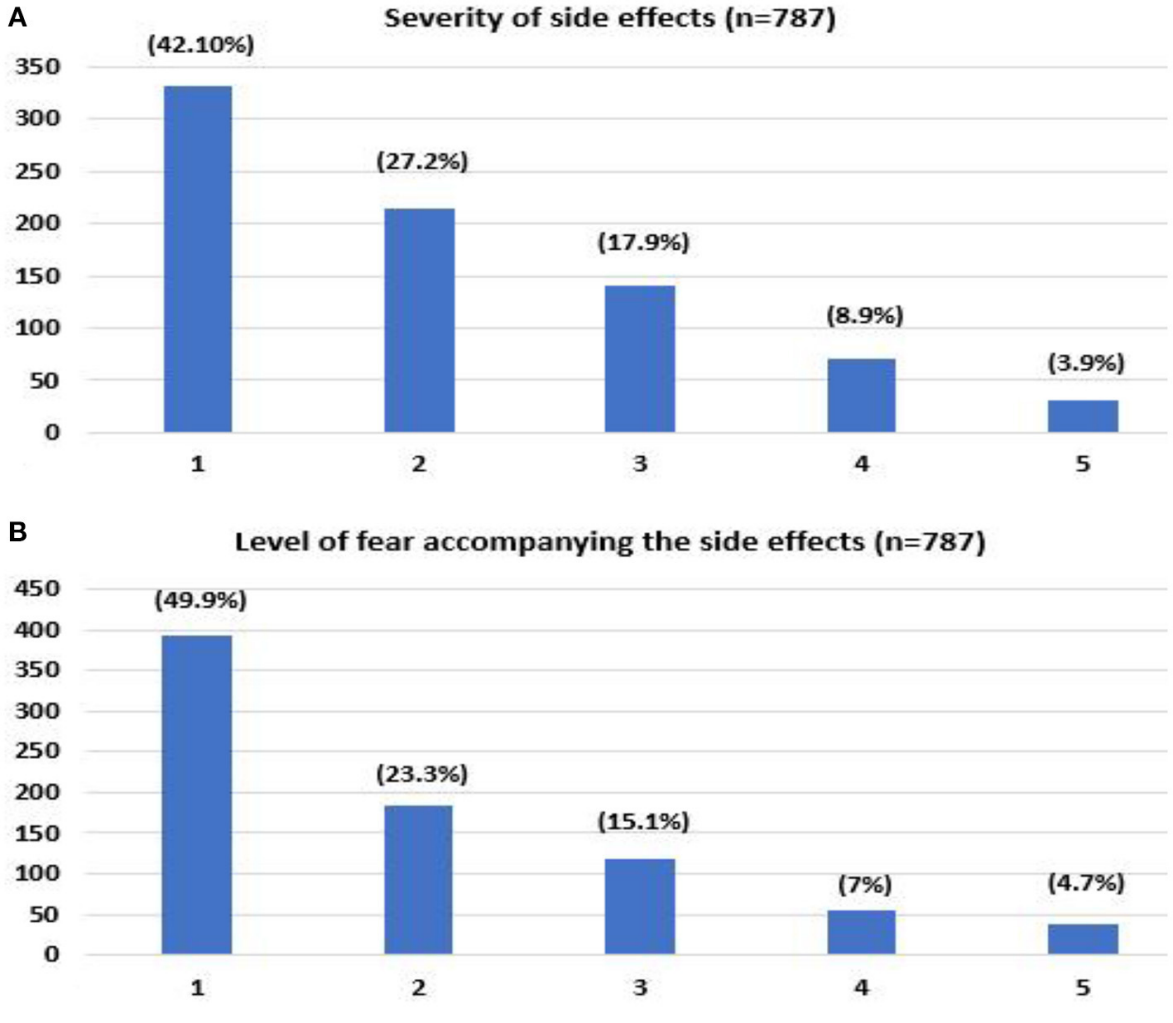
Self-reported severity of side effects that occurred after receiving COVID-19 vaccine **(A)**, level of fear associated with previous COVID-19 vaccine **(B)**. (1- no/negligible, 5- severe).

The majority of the respondents reported no/negligible side effects after the initial vaccine doses (42.1%), as shown in Figure 1A and Table 2. As to the fear associated with receiving the vaccine, respondents predominantly expressed an absence or low level of fear for previous doses (49.9%) (Figure 1B and Table 2).

**Table 2:** Characteristics of the respondents.

**Variables**	**Characteristics**	**Frequency**	**Percentage**
Severity of side-effects that occurred after receiving COVID-19	1	331	42.1
vaccine (1- no/negligible side effects, 5-severe side effects)	2	214	27.2
	3	141	17.9
	4	70	8.9
	5	31	3.9
Level of fear accompanying side effects after receiving COVID-19	1	393	49.9
vaccine (1- no/very low fear, 5- very high fear)	2	183	23.3
	3	119	15.1
	4	55	7.0
	5	37	4.7
Comorbidities	Diabetes	12	1.5
	Hypertension	29	3.7
	Cancer	6	0.8
	Cardiovascular disease	6	0.8
	Chronic pulmonary disease	8	1.0
	Chronic kidney disease	5	0.6
	Asthma	65	8.3
	None of these	693	88.1

### Willingness to Receive a Booster COVID-19 Vaccine Dose

**Figure 2:**
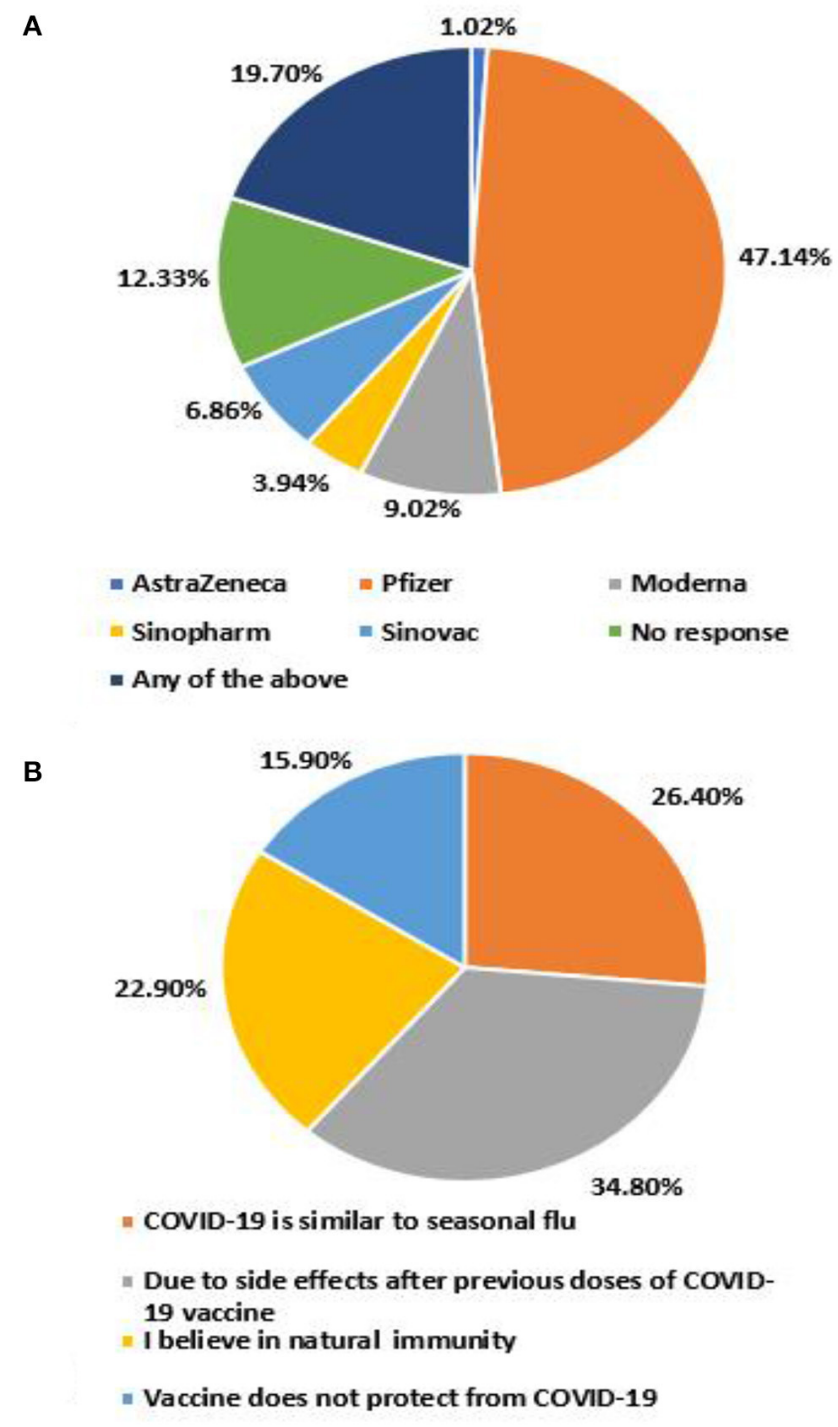
**(A)** Reasons of unwillingness, **(B)** choice of booster dose.

Overall, 77.8% of the participants were willing to receive the COVID-19 vaccine booster dose. The chief reasons for unwillingness were safety concerns due to side effects after previous doses (34.8%), COVID- 19 being similar to seasonal flu (26.4%), followed by belief in natural immunity (22.9%) (Figure 2A). 47.1% reported the preference for BioNTech-Pfizer vaccine as the booster, whereas AstraZeneca was the least preferred (1.0%) (Figure 2B).

### Self-Perceived Side Effects, Fear and Attitudes Regarding Boosters

**Figure 3:**
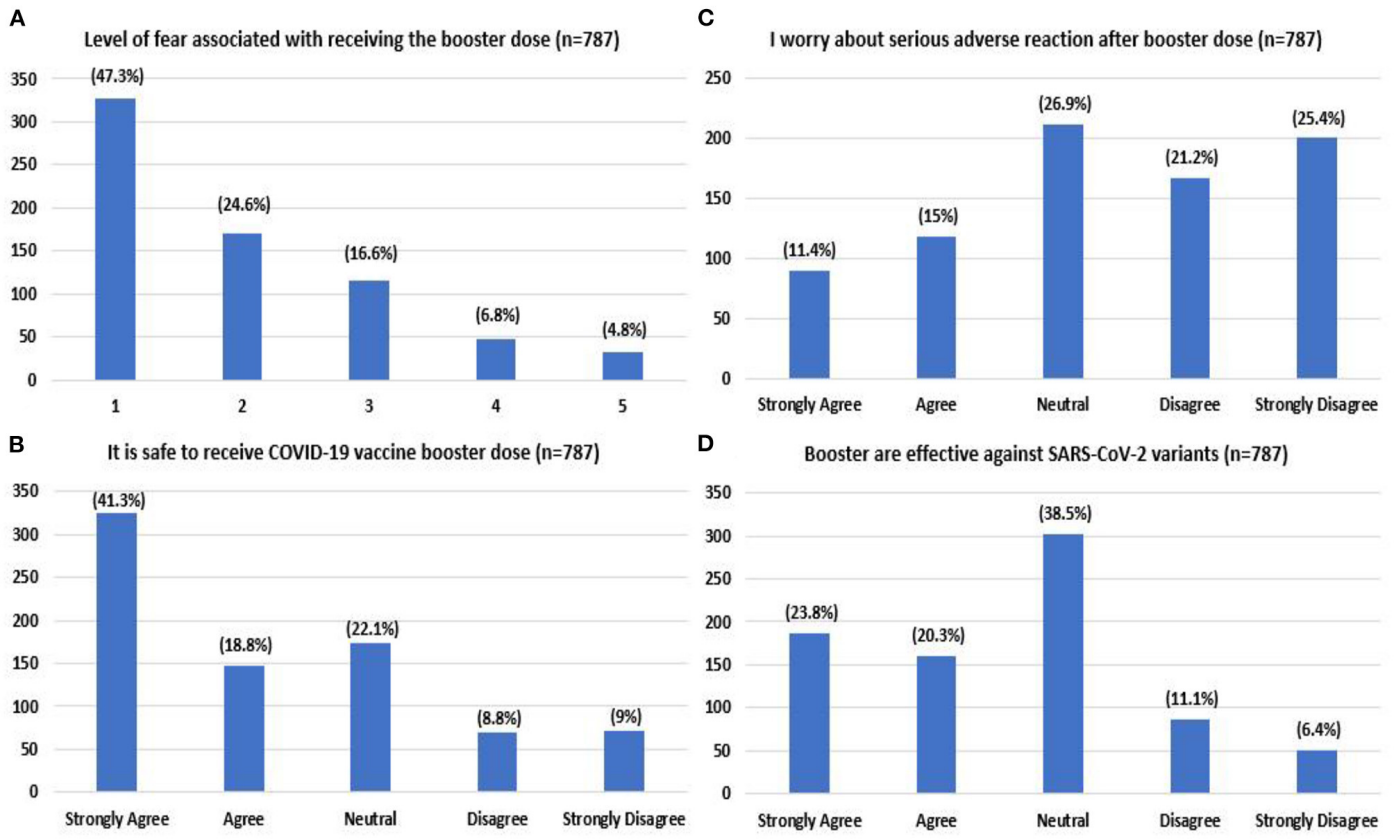
Level of fear associated with booster dose **(A)** (1- no/very low fear, 5- very high fear) attitude toward booster dose regarding level of safety **(B)**, worry **(C)**, and effectiveness **(D)** reported by the study participants.

Respondents for the most part, expressed an absence or low level of fear for the booster dose (47.3%) (Figure 3A). Most respondents agreed/strongly agreed that it was safe to receive an additional dose (60.1%). Few agreed/strongly agreed with the statement that they were worried about severe adverse reactions after the additional dose (26.4%). Regarding the vaccine's efficacy, 44.1% strongly agreed/agreed that boosters are effective against COVID-19 variants (Figure 3).

### Factors Associated With Booster Acceptance

Household income ranging between 100,000 and 200,000 PKR (*p* = 0.025) and 200,000 and 300,000 PKR (*p* = 0.006) was associated with a significantly higher willingness for a booster shot. Absence of comorbidities (*p* = 0.047) and little to no side effects after previous doses [levels 1 (*p* < 0.001) and 2 (*p* = 0.003)] also increased vaccine willingness, significantly. Additionally, lower levels of fear (1, 2, 3, and 4) for vaccine and booster doses were significantly associated with an increased willingness (*p* < 0.05). Unwillingness to pay for the booster (*p* < 0.001) and partial vaccination (*p* = 0.023) were significantly associated with increased hesitancy. Study participants who gave no response for the level of fear associated with booster were less willing to receive the additional dose (*p* < 0.001; Table 3).

**Table 3:** Univariable analysis of study variables to willingness of booster dose.

**Variables**	**Willing to receive** **booster dose**	**Not willing to receive/Unsure**	**OR (95% confidence interval)**	***p*-value**
	**(*n* = 612)**	**(*n* = 175)**		
**Age**				
<18 years	103 (16.8%)	39 (22.3%)	0.629 (0.288–1.374)	0.245
18-20 years	208 (34.0%)	51 (29.1%)	0.971 (0.457–2.065)	0.939
21-30 years	259 (42.3%)	75 (42.9%)	0.822 (0.394–1.176)	0.602
>30 years	42 (6.9%)	10 (5.7%)	Ref	-
**Gender**				
Female	419 (68.5%)	129 (73.7%)	0.774 (0.531–1.129)	0.184
Male	193 (31.5%)	46 (26.3%)	Ref	-
**Education**				
Primary/Secondary	28 (4.6%)	11 (6.3%)	Ref	-
Higher secondary/College	203 (33.2%)	67 (38.3%)	1.190 (0.562–2.520)	0.649
Undergraduate/Bachelors/University	332 (54.2%)	89 (50.9%)	1.465 (0.702–3.058)	0.309
Postgraduate/Masters/Ph.D.	49 (8.0%)	8 (4.6%)	2.406 (0.866–6.688)	0.092
**Employment**				
Employed	70 (11.4%)	16 (9.1%)	1.260 (0.710–2.237)	0.430
Self-employed	42 (6.9%)	15 (8.6%)	0.806 (0.435–1.496)	0.495
Unemployed	500 (81.7%)	144 (82.3%)	Ref	-
**Household income (per month)**				
<50,000 PKR	93 (15.2%)	36 (20.6%)	Ref	-
50,000–100,000 PKR	130 (21.2%)	46 (26.3%)	1.094 (0.656–1.824)	0.730
100,000–200,000 PKR	137 (22.4%)	28 (16.0%)	1.894 (1.082–3.315)	0.025
200,000–300,000 PKR	80 (13.1%)	11 (6.3%)	2.815 (1.345–5.891)	0.006
300,000–400,000 PKR	57 (9.3%)	13 (7.4%)	1.697 (0.830–3.469)	0.147
400,000–500,000 PKR	24 (3.9%)	9 (5.1%)	1.032 (0.438–2.433)	0.942
>500,000 PKR	91 (14.9%)	32 (18.3%)	1.101 (0.631–1.921)	0.735
**Status of vaccination**				
Fully vaccinated	596 (97.4%)	164 (93.7%)	Ref	-
Partially vaccinated	16 (2.6%)	11 (6.3%)	0.400 (0.182–0.879)	0.023
**Previously infected with SARS-CoV-2**				
Yes	178 (29.1%)	49 (28.0%)	Ref	-
No	434 (70.9%)	126 (72.0%)	0.948 (0.653–1.377)	0.780
**Willingness to pay for booster dose**				
Yes	389 (63.6%)	45 (25.7%)	Ref	
No	223 (36.4%)	130 (74.3%)	0.198 (0.136–0.289)	<0.001
**Presence of comorbidities**				
Yes	67 (10.9%)	29 (16.6%)	Ref	
No	545 (89.1%)	146 (83.4%)	1.616 (1.007–2.591)	0.047
**Severity of side effects that occurred after receiving COVID-19 vaccine**				
No/negligible side effects	274 (44.8%)	57 (32.6%)	3.959 (1.846–8.488)	<0.001
Low side effects	171 (27.9%)	43 (24.6%)	3.275 (1.498–7.161)	0.003
Mild side effects	98 (16.0%)	43 (24.6%)	1.877 (0.849–4.148)	0.120
Moderate side effects	52 (8.5%)	18 (10.3%)	2.379 (0.979–5.779)	0.056
Severe side effects	17 (2.8%)	14 (8.0%)	Ref	-
**Level of fear accompanying while receiving COVID-19 vaccine**				
No/very low level of fear	334 (54.6%)	59 (33.7%)	10.451 (5.039–21.676)	<0.001
Low fear	151 (24.7%)	32 (18.3%)	8.712 (4.012–18.914)	<0.001
Neutral	80 (13.1%)	39 (22.3%)	3.787 (1.743–8.228)	0.001
High fear	34 (5.6%)	21 (12.0%)	2.989 (1.256–7.112)	0.013
Very high fear	13 (2.1%)	24 (13.7%)	Ref	-
**Level of fear associated with**				
**receiving potential additional dose of COVID-19 vaccine**				
No/very low level of fear	310 (50.7%)	18 (10.3%)	20.667 (8.977–47.578)	<0.001
Low fear	158 (25.8%)	13 (7.4%)	14.585 (5.999–35.460)	<0.001
Neutral	93 (15.2%)	22 (12.6%)	5.073 (2.217–11.609)	<0.001
High fear	33 (5.4%)	14 (8.0%)	2.829 (1.119–7.151)	0.028
Very high fear	15 (2.5%)	18 (10.3%)	Ref	-
Prefer not to say/no response	3 (0.5%)	90 (51.4%)	0.040 (0.010–0.153)	<0.001

*PKR, Pakistani Rupee; SARS-CoV-2, severe acute respiratory syndrome coronavirus 2; Ph.D., Doctor of Philosophy; OR, odds ratio; COVID-19, coronavirus disease 2019; Ref, reference category*.

**Figure 4:**
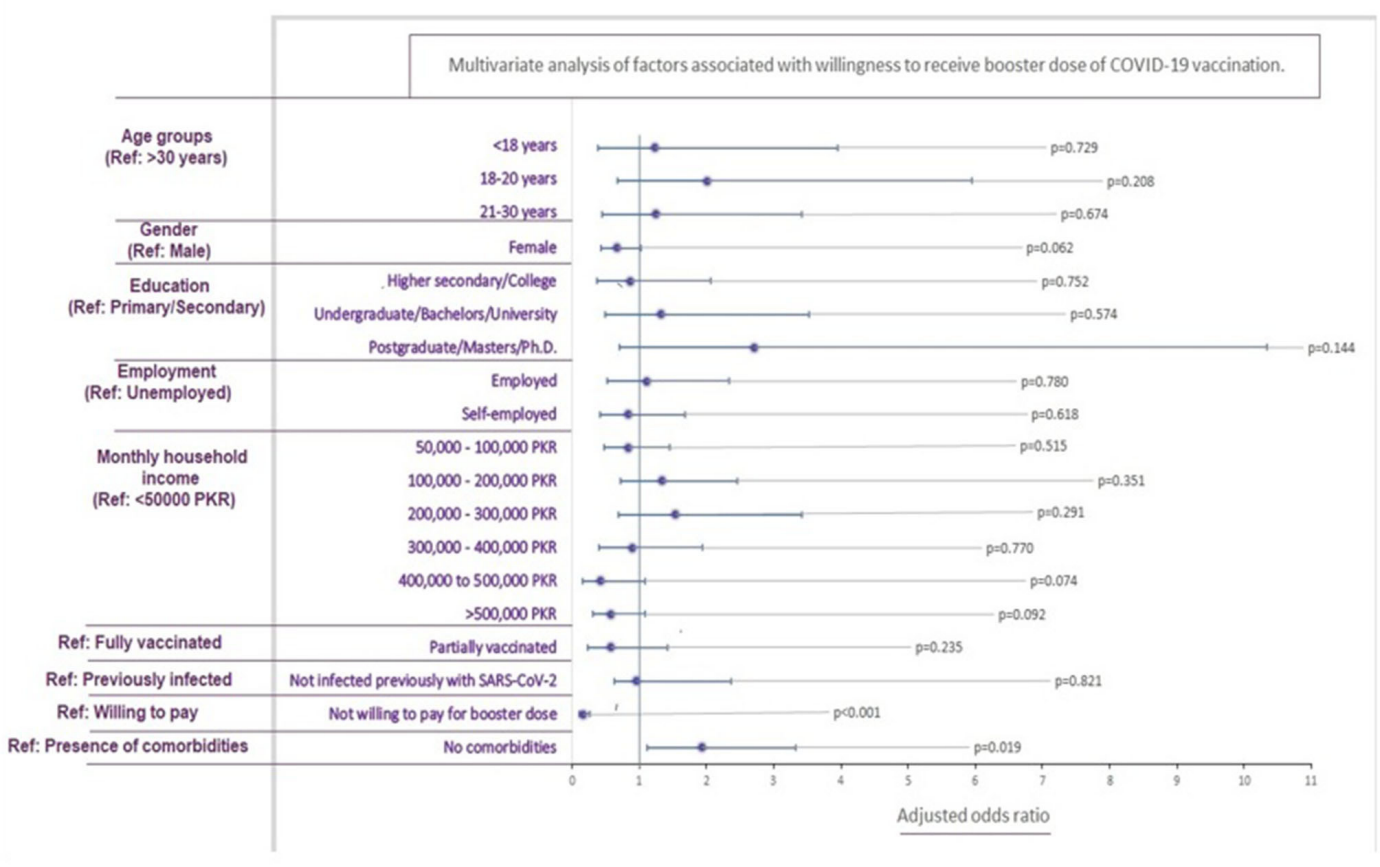
Multivariable analysis of factors associated with willingness to receive booster dose.

Upon multivariable analysis, only the absence of comorbidities was associated significantly with increased booster willingness (*p* = 0.019). Contrarily unwillingness to pay for the booster was associated with increased hesitancy (*p* < 0.001; Figure 4).

## Discussion

The advent of COVID-19 vaccines has significantly improved immunity and mortality rates while preventing hospitalizations in immunocompromised individuals. A recent study confirms that the neutralization offered by any vaccine's first two doses declines over 20 weeks after the second dose (25). COVID-19 vaccines were least effective against the latest mutation of the SARS-CoV-2 virus, the Omicron (B.1.1.529) variant, requiring a third or a booster dose of the COVID-19 vaccine 6 months after the two- dose regime. Following the evidence of waning protection and the fifth wave of the coronavirus, many countries, including Pakistan's National Command Operation Centre (NCOC), made it compulsory on December 1st, 2021, for healthcare workers, immunocompromised individuals, and people aged > 50 to receive a booster shot of either BNT162b or mRNA-1273 (26). It was not until January 3rd, 2022, that individuals over 30 could receive a booster shot (27). Only 4.8% of the vaccinated population in Pakistan (28) has received a booster dose, compelling us to study the willingness and perceptions against the COVID-19 booster dose. This is the first cross-sectional survey that assesses willingness and the influencing factors of COVID-19 vaccine booster hesitancy (VBH) and barriers, using a random sample of the vaccinated population in Pakistan.

A high proportion of the participants, approximately 78%, reflected decreased hesitancy to receive a COVID-19 booster dose. Despite the positive attitudes, a 22% COVID-19 booster dose hesitancy should be alarming for the frail healthcare sector of Pakistan. Although these results are comparable with surveys conducted in China and Poland, with COVID-19 booster hesitancy varying between 5 and 30% (29–32), it is imperative to note that some of the studies were conducted prior to the official approval of COVID-19 boosters. An online survey from Algeria reflects similar hesitancy percentages (23%) in the general population; however, 25% rejected the COVID-19 booster (33). The lack of empirical evidence on COVID-19 booster acceptance from more developing or Asian countries restricts us from drawing effective comparisons with prior studies. However, a cross-sectional survey between December 2020 and March 2021 in Pakistan revealed that 35% of 1,014 participants were unwilling to receive the two-dosage regime of the COVID-19 vaccine (34).

A univariate regression analysis was performed to determine the association of sociodemographic characteristics, fear, and previous medical history on COVID-19 booster acceptance. Respondents with a monthly household income between PKR 100,000 to 300,000 (USD 550–1,650) demonstrated low hesitance (*P* < 0.001) to receive the booster shot. Our results differ slightly from a previous study in Pakistan (20), which concluded positive COVID-19 vaccine acceptance in households with monthly family income > PKR 50,000, reflecting low education and awareness levels influencing decisions in individuals with low socioeconomic status strata. However, in a country with increasing inflation and poverty rates, a decreased booster acceptance in such households merits attention to removing financial barriers and constraints. Contrarily, the controversies and myths could significantly influence a higher socioeconomic background due to increased exposure to social media (35).

Other sociodemographic characteristics, such as age, sex, and education level, were unrelated to COVID- 19 vaccine booster hesitancy. A varying trend of willingness was observed with age. Booster acceptance was most remarkable in the 18–20 group (80.3%) and >30 age group (80.7%). The greatest hesitancy was observed in the under 18 age group (72.5%), which may include teens with little understanding of the efficacy of the COVID-19 vaccine. Our findings differ from studies by Qin et al. and Folcarelli et al. The 21–30 age group (31) and the older participants (36) demonstrated the lowest hesitancy, respectively. Consistent with evidence from existing literature (29), females were reluctant to receive the booster dose. Social media reports demonstrate disturbances in the menstrual cycle. A recent study (37) concluded that COVID-19 vaccination is associated with a small menstrual cycle length. Additionally, females have been vastly underrepresented in vaccine trials, explaining their hesitancy.

Based on our study, the primary predictor of booster unwillingness was the safety concern against the COVID-19 vaccines (44.3%), comparable with previous studies' results (34, 38). This could originate from the fear associated with potential side effects of a COVID-19 booster dose, as revealed by this study, in which 53% of 693 respondents expressed moderate to high levels of fear. However, lower levels of fear were associated (*P* < 0.05) with decreased booster hesitancy. When assessing attitudes regarding the booster dose, 26.4% of individuals in the study strongly expressed their worry about severe adverse reactions from the booster dose. Commonly reported local and systemic side effects with any COVID-19 vaccine include fever, headache, myalgia, nausea, vomiting and fatigue. These symptoms are mild to moderate in intensity and resolve within a few days of the vaccine (39). Initial reports of cardiac and vascular manifestations, such as myocarditis, thrombotic and embolic events from mRNA COVID-19 vaccines (40–42), have instilled fear. A systematic review of vaccine hesitancy in the US revealed that a lack of education and understanding about vaccine development merits vaccine hesitancy in the general population (43). Additionally, the Pakistani population tends to believe in conspiracy theories such as infertility related to COVID-19 vaccines, reflecting their lack of confidence in medical providers and the healthcare system (44). In association with the Ministry of Information, Pakistan Electronic Media Regulatory Authority (PEMRA) should control the airing of controversial and unsupported theories against the COVID-19 booster dose (45).

88.1% of the participants did not present with chronic health issues like diabetes, hypertension, and cardiovascular diseases. Univariate analysis of healthy participants expressed a positive attitude (*P* = 0.047) to receive a COVID-19 vaccine booster, with the multivariate analysis producing a similar result (*P* = 0.019). As discussed previously, reports of adverse events and high morbidity and mortality (46) have instilled fear. This is likely to give rise to mistrust among vulnerable chronic disease patients, requiring a cautious approach to vaccination. Our results differ from the findings by Paul et al., which revealed that healthy individuals were uncertain to receive a booster dose (47). This is likely a result of misinformation that natural immunity confers complete protection from the coronavirus infection. Similarly, studies from Indonesia and Poland revealed that immunosuppressive patients were willing to receive the additional COVID-19 booster dose, to prevent serious complications (21, 48).

A large group (22.9%) of participants in this cross-sectional survey believed that natural immunity was sufficient in protecting against SARS-CoV-2. This could potentially originate from the belief that natural immunity increases after a two-dose regime of the COVID-19 vaccine or the belief that acquired immunity from a previous COVID-19 infection confers sufficient protection (34). 16.9% of respondents in our study believed that SARS-CoV-2 was similar to the seasonal flu, with low infection risk, therefore posing a limited threat. Given that this survey was carried out during the fifth wave of the highly virulent omicron coronavirus strain, a group of vaccinated participants (15.9%) expressed that the vaccines do not confer long-last protection from COVID-19. Our study also revealed that 56% of the participants had a neutral stance or perceived inefficacy of the vaccine booster against the SARS-CoV-2 variants. This requires the NCOC to strategize the COVID-19 booster regime and spread awareness regarding the effectiveness of booster dosages.

Of the 787 participants of this study, 37 had received only a single dose of the two-dose COVID-19 vaccine, which was a significant predictor of COVID-19 booster hesitancy (*P* = 0.023). Although a multivariate analysis produced nonsignificant results (*P* = 0.235), partial COVID-19 vaccination threatens Pakistan's health sector following an arduous history of inoculation drives such as polio, which has received nationwide reluctance (35). Amidst the rise of the delta-variant COVID-19 cases, the government saw a surge in vaccine percentages after it had banned telecommunication services and withheld salaries of unvaccinated Pakistanis (49). Similar approaches could be beneficial in overcoming booster dose hesitancy.

This study identifies a potential barrier to any vaccine drive in Pakistan, owing to vaccine selectivity, which is defined as discriminatory attitudes toward certain types of vaccines. This survey revealed that 19.7% were willing to receive any available COVID-19 vaccine. 47.6% of the respondents expressed willingness to receive a booster dose of the BNT162b2 (BioNTech-Pfizer) vaccine, followed by 10% of participants willing to receive mRNA-1273 (Moderna) or ChAdOx1-S/nCoV-19 (AstraZeneca); only a small fraction (11%) preferred CoronaVac (Sinovac) or BBIBP-CorV (Sinopharm) COVID-19 vaccine. Our findings align with the previous study (34), in which participants preferred a vaccine originating from the UK and the US as opposed to China. The uncertainty and mistrust could be due to the travel bans on Pakistani travelers who had only received COVID-19 vaccines of Chinese origin (50), giving rise to disparity amongst low and high socioeconomic countries.

The most significant contribution to the COVID-19 vaccination drive in Pakistan has been by the World Health Organization, under the COVAX agreement (51). Given the limited number of vaccines for a growing population, in September 2021, the Pakistani government fixed PKR 1,250 (USD 7) for the COVID-19 booster dose (52). Additionally, the booster dose was initially only available for travelers, becoming a source of misinformation amongst the general Pakistani population. In our survey, 74.3% of participants were reluctant to pay for the dose, with the results remaining significant (*P* < 0.001) with univariate and multivariate analysis.

Given the limited resources, the Pakistani government's efforts to increase vaccine coverage have been commendable. With one of the lowest adult literacy rates, 63%, in 2021 (49), Pakistan has introduced a coronavirus public service message on caller tunes and televisions. While temporarily impactful, the Expanded Program on Immunization, in collaboration with WHO, can address the anti-vaccination drives in the future.

Although this cross-sectional survey was the first to explore the predictors of COVID-19 booster hesitancy in this region of South Asia, it is prone to some limitations. Since the questionnaire was distributed on online platforms, selection bias could be related to limited participants having access to the internet or social media. Social media could also affect their opinions and intent to receive the COVID-19 booster. Individuals likely have extreme views on the spectrum, and therefore, it does not reflect the attitudes and beliefs of the entire nation. This survey has a significant representation of females but lacks responses from different ethnicity and occupational subgroups. The majority of the respondents in this study belonged to the age group 18–30 years, depicting a population of students. Thus, the high unemployment rate is not a representation of the actual situation in Pakistan. It also did not account for chronic mental illnesses in the sample population. The survey was conducted when the coronavirus wave was relatively stable, influencing the individuals' decision to vaccinate.

## Conclusion

The current cross-sectional survey yields an alarming result, with 22.2% of individuals expressing COVID-19 vaccine booster hesitancy in the Pakistani population. Participants expressed concerns about booster safety, with 43.7% fearing side effects. The exisiting comorbidities amongst the respondents and the notion that perceived natural immunity protects against the coronavirus contributed significantly to decreased booster willingness. Given the general population's history of reluctance toward immunization drives, public health policymakers must undertake necessary awareness campaigns to strategize vaccination distribution.

## Data Availability Statement

The raw data supporting the conclusions of this article will be made available by the authors, without undue reservation.

## Author Contributions

AM contributed to the design, conception, and review of the study. HN reviewed the manuscript and contributed to the manuscript writing. AS designed the questionnaire and interpreted the results. MA performed the statistical analysis. HR contributed in manuscript writing. AK contributed by collecting responses. ZB helped with the manuscript writing. BS reviewed and finalized the manuscript. All authors listed have made a substantial, direct, and intellectual contribution to the work and approved it for publication.

## Conflict of Interest

The authors declare that the research was conducted in the absence of any commercial or financial relationships that could be construed as a potential conflict of interest.

## Publisher's Note

All claims expressed in this article are solely those of the authors and do not necessarily represent those of their affiliated organizations, or those of the publisher, the editors and the reviewers. Any product that may be evaluated in this article, or claim that may be made by its manufacturer, is not guaranteed or endorsed by the publisher.
